# ADAM10 Releases a Soluble Form of the GPNMB/Osteoactivin Extracellular Domain with Angiogenic Properties

**DOI:** 10.1371/journal.pone.0012093

**Published:** 2010-08-10

**Authors:** April A. N. Rose, Matthew G. Annis, Zhifeng Dong, Francois Pepin, Michael Hallett, Morag Park, Peter M. Siegel

**Affiliations:** 1 Goodman Cancer Research Centre, McGill University, Montreal, Quebec, Canada; 2 Department of Medicine, McGill University, Montreal, Quebec, Canada; 3 Department of Biochemistry, McGill University, Montreal, Quebec, Canada; 4 Department of Oncology, McGill University, Montreal, Quebec, Canada; 5 Life Science Division, Lawrence Berkeley National Laboratory, Berkeley, California, United States of America; 6 McGill Centre for Bioinformatics, McGill University, Montreal, Quebec, Canada; Indiana University, United States of America

## Abstract

**Background:**

Glycoprotein non-metastatic melanoma protein B (GPNMB)/Osteoactivin (OA) is a transmembrane protein expressed in approximately 40–75% of breast cancers. GPNMB/OA promotes the migration, invasion and metastasis of breast cancer cells; it is commonly expressed in basal/triple-negative breast tumors and is associated with shorter recurrence-free and overall survival times in patients with breast cancer. Thus, GPNMB/OA represents an attractive target for therapeutic intervention in breast cancer; however, little is known about the functions of GPNMB/OA within the primary tumor microenvironment.

**Methodology/Principal Findings:**

We have employed mouse and human breast cancer cells to investigate the effects of GPNMB/OA on tumor growth and angiogenesis. GPNMB/OA-expressing tumors display elevated endothelial recruitment and reduced apoptosis when compared to vector control-derived tumors. Primary human breast cancers characterized by high vascular density also display elevated levels of GPNMB/OA when compared to those with low vascular density. Using immunoblot and ELISA assays, we demonstrate the GPNMB/OA ectodomain is shed from the surface of breast cancer cells. Transient siRNA-mediated knockdown studies of known sheddases identified ADAM10 as the protease responsible for GPNMB/OA processing. Finally, we demonstrate that the shed extracellular domain (ECD) of GPNMB/OA can promote endothelial migration *in vitro*.

**Conclusions/Significance:**

GPNMB/OA expression promotes tumor growth, which is associated with enhanced endothelial recruitment. We identify ADAM10 as a sheddase capable of releasing the GPNMB/OA ectodomain from the surface of breast cancer cells, which induces endothelial cell migration. Thus, ectodomain shedding may serve as a novel mechanism by which GPNMB/OA promotes angiogenesis in breast cancer.

## Introduction

Glycoprotein non-metastatic melanoma protein B (GPNMB) is a type I transmembrane protein that is also known as Osteoactivin (OA), Dendritic Cell–Heparin Integrin Ligand (DC-HIL) or Hematopoietic Growth Factor Inducible Neurokinin-1 type (HGFIN). GPNMB/OA is expressed in a wide array of normal tissue types including: the bone, hematopoietic system and the skin. Within the bone, GPNMB/OA has been shown to promote the differentiation of both osteoclasts [1], [2] and osteoblasts [3], [4]. GPNMB/OA is also readily detectable in immune cells, such as macrophages and dendritic cells [5], [6], and has been shown to functionally impair T-cell activation [7], [8]. Within the skin, GPNMB/OA has been proposed to be expressed specifically in melanocytes [9], while others suggest a broader pattern of expression that includes keratinocytes, melanocytes and Langerhans cells [7].

In addition to its diverse roles in normal cells, aberrant GPNMB/OA expression has been linked to various pathological disorders such as glaucoma [10], kidney disease [11], osteoarthritis [12] and several types of cancer, including: uveal melanoma [13], glioma [14], [15], hepatocellular carcinoma [16] and cutaneous melanoma [17]. Recently, we demonstrated that GPNMB/OA is highly expressed in several aggressively bone-metastatic sub-populations of the 4T1 mouse mammary carcinoma cell line. Moreover, we showed that ectopic expression of GPNMB/OA in poorly metastatic 66cl4 mouse mammary carcinoma cells is sufficient to induce MMP-3 expression and increases their invasion *in vitro* and promotes bone metastasis *in vivo* [18]. Subsequently, we employed IHC-based analysis of tissue microarrays to investigate the relevance of GPNMB/OA expression in human breast cancer, and found that GPNMB/OA is expressed in the tumor epithelium of approximately 10% of human breast cancers and the stromal compartment of nearly 70% of breast tumors. Moreover, epithelial, but not stromal, GPNMB/OA expression is a prognostic indicator of cancer recurrence across all breast cancer subtypes, and specifically within “triple negative” breast cancers [19].

GPNMB/OA is localized to diverse subcellular locations within the cell, including the plasma membrane of cancer cells [17], [19], within melanosomes of melanoma cells [7] and within endocytic/lysosomal vesicles in osteoclasts [1]. Two *GPNMB/OA* mRNA isoforms encoding 560 and 572 amino acid proteins have been identified; the longer isoform corresponds to a splice variant that contains an in-frame 12 amino acid insertion within the extracellular domain [14]. Both isoforms contain a large extracellular domain (ECD), a single pass transmembrane domain and a short cytoplasmic tail. The GPNMB/OA ECD contains an integrin-binding RGD domain that is required for the GPNMB/OA-dependent adhesive interaction between melanocytes and keratinocytes [7] and a polycystic kidney disease (PKD) domain whose function in GPNMB/OA remains unknown. Moreover, several groups have reported that GPNMB/OA is proteolytically cleaved in an MMP-dependent manner [9], [20], [21]. Interestingly, NIH-3T3 fibroblasts stimulated with a recombinant GPNMB/OA ECD displayed enhanced Erk and p38 phosphorylation along with the upregulation of *Mmp-3* mRNA [20].

Given the utility of GPNMB/OA expression as a prognostic indicator of recurrence and its potential as a therapeutic target in human breast tumors [22], [23], we aimed to investigate the functional role of GPNMB/OA in the primary breast tumor microenvironment. We demonstrate that GPNMB/OA expression enhances primary tumor growth, which is associated with diminished apoptosis and elevated recruitment of endothelial cells. GPNMB/OA is constitutively shed from breast cancer cells in an ADAM10-dependent manner and the shed GPNMB/OA ECD is capable of inducing endothelial cell migration *in vitro*. Thus, we are the first to implicate ADAM10 as a sheddase that liberates GPNMB/OA ECD and to describe a functional role for the GPNMB/OA ECD in promoting endothelial cell migration.

## Results

### Ectopic GPNMB/OA expression enhances primary tumor growth

**Figure 1:**
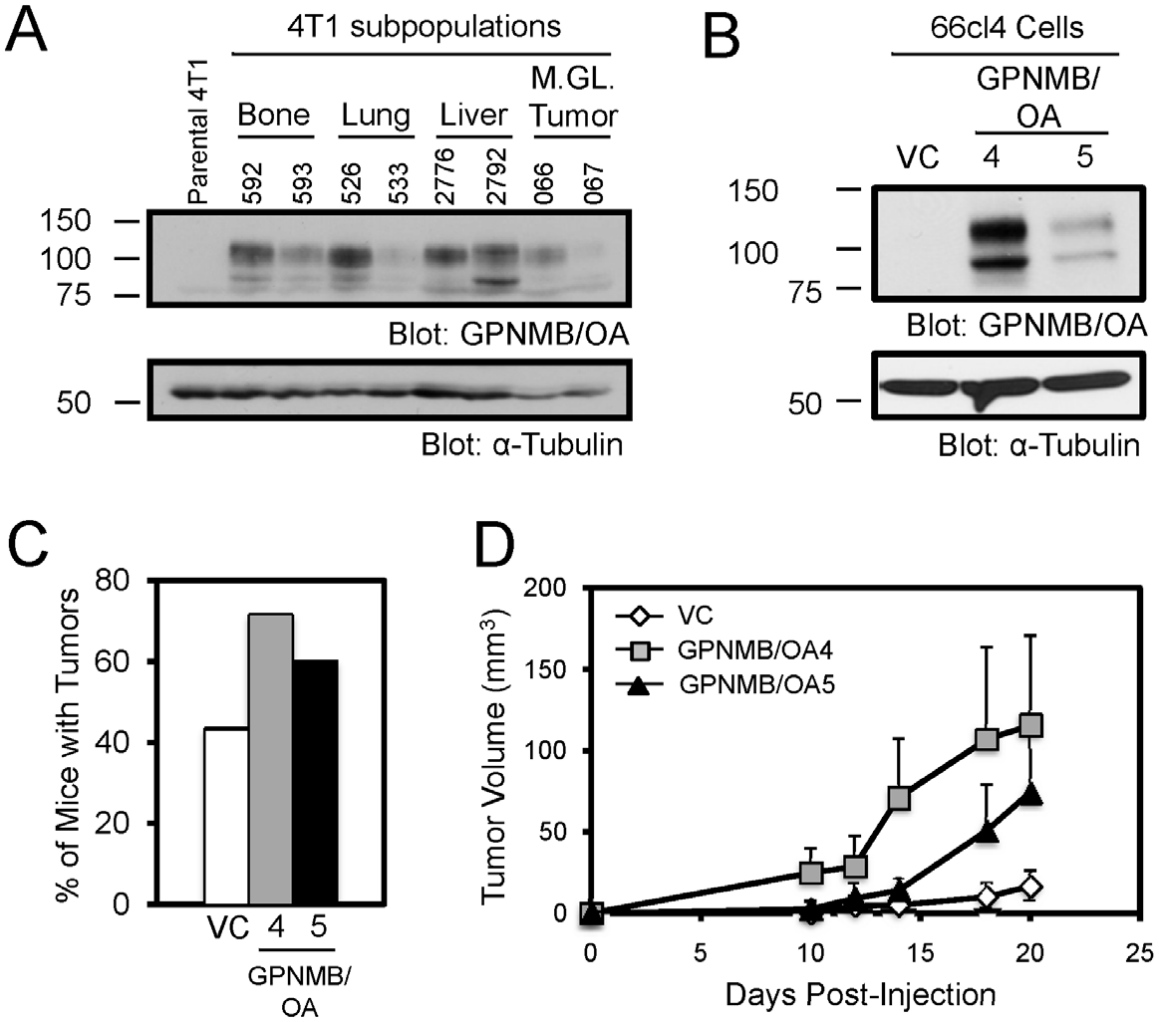
GPNMB/OA enhances primary tumor growth. (**A**) Immunoblot analysis of GPNMB/OA expression in parental cells (4T1) and explants taken from primary tumors (066, 067) and the following metastatic sites: bone (592, 593), lung (526, 533) and liver (2776, 2792). (**B**) Expression of GPNMB/OA was confirmed by immunoblot analysis of total cell lysates from vector control (VC) and two clonal cell lines expressing GPNMB/OA (GPNMB/OA4 and GPNMB/OA5). As a loading control, total cell lysates were blotted for α-Tubulin (**A**, **B**). (**C**) Percentage of Balb/c mice that developed mammary tumors reaching 200mm^3^ by 6 weeks post-injection of VC (n = 13/30), GPNMB/OA4 (n = 20/28) or GPNMB/OA5 (n = 6/10) expressing 66cl4 cells. (**D**) Tumor growth curves in mice injected with VC (n = 13), GPNMB/OA4 (n = 20) and GPNMB/OA5 (n = 6) expressing 66cl4 cells.

Previously we have reported that GPNMB/OA expression is increased in *in vivo* selected aggressively bone metastatic subpopulations of 4T1 mammary carcinoma cells [18]. In addition to bone metastatic sub-populations (592, 593), GPNMB/OA is also overexpressed in 4T1 sub-populations that are either aggressively metastatic to lung (526), liver (2776, 2792) or that have been explanted from primary tumors (066) (**Figure 1A**). This is consistent with our previous observations that GPNMB/OA is also overexpressed in human breast tumors, and suggests that GPNMB/OA may be functionally implicated in regulating tumor growth in addition to promoting invasion and metastasis [18], [19]. To investigate this hypothesis, we employed an independent, less aggressive mammary tumor cell line in which we generated one pooled vector control (VC), and two clonal populations (GPNMB/OA4, GPNMB/OA5) of 66cl4 mouse mammary carcinoma cells. Variable levels of GPNMB/OA protein could be detected in the cell lysates of 66cl4-OA4 and 66cl4-OA5 cells (**Figure 1B**). To assess the consequences of GPNMB/OA expression on primary mammary tumor growth, 66cl4 cells were injected into the mammary fat pads of Balb/c mice. GPNMB/OA increased the incidence of mammary tumor formation (**Figure 1C**) and also accelerated tumor outgrowth relative to VC tumors (**Figure 1D**). Moreover, the kinetics of tumor outgrowth correlated with the level of GPNMB/OA expressed in these cells (**Figure 1B, D**). To rule out the possibility that these findings reflect phenotypes associated with clonal breast cancer populations, we generated a population of pooled GPNMB/OA expressing cells (**Supplemental Figure S1A**) and found that these too enhanced tumor growth relative to vector control cells (**Supplemental Figure S1B**).

GPNMB/OA expressed on antigen presenting cells can suppress T-cell activation [7], [8], [24]. Recently, it has been shown that GPNMB/OA expressed in melanoma cells promotes their growth by impairing the activation of melanoma-reactive T-cells [25]. To assess whether a similar mechanism could account for GPNMB/OA-induced mammary tumor growth observed in Balb/c mice, we performed a second set of mammary fat pad injections into athymic mice that lack functional T-cells. Importantly, the GPNMB/OA-associated increase in tumor outgrowth observed in Balb/c mice was maintained even when cells were injected into immunodeficient mice, although to a lesser degree when compared to injections performed in Balb/c mice (**Supplemental Figure S1B**). Thus, the tumor growth promoting effects of GPNMB/OA cannot be fully explained through a mechanism involving suppression of T-cell activation.

### GPNMB/OA expression in breast cancer cells is associated with decreased apoptosis and increased vascular density in vivo

**Figure 2:**
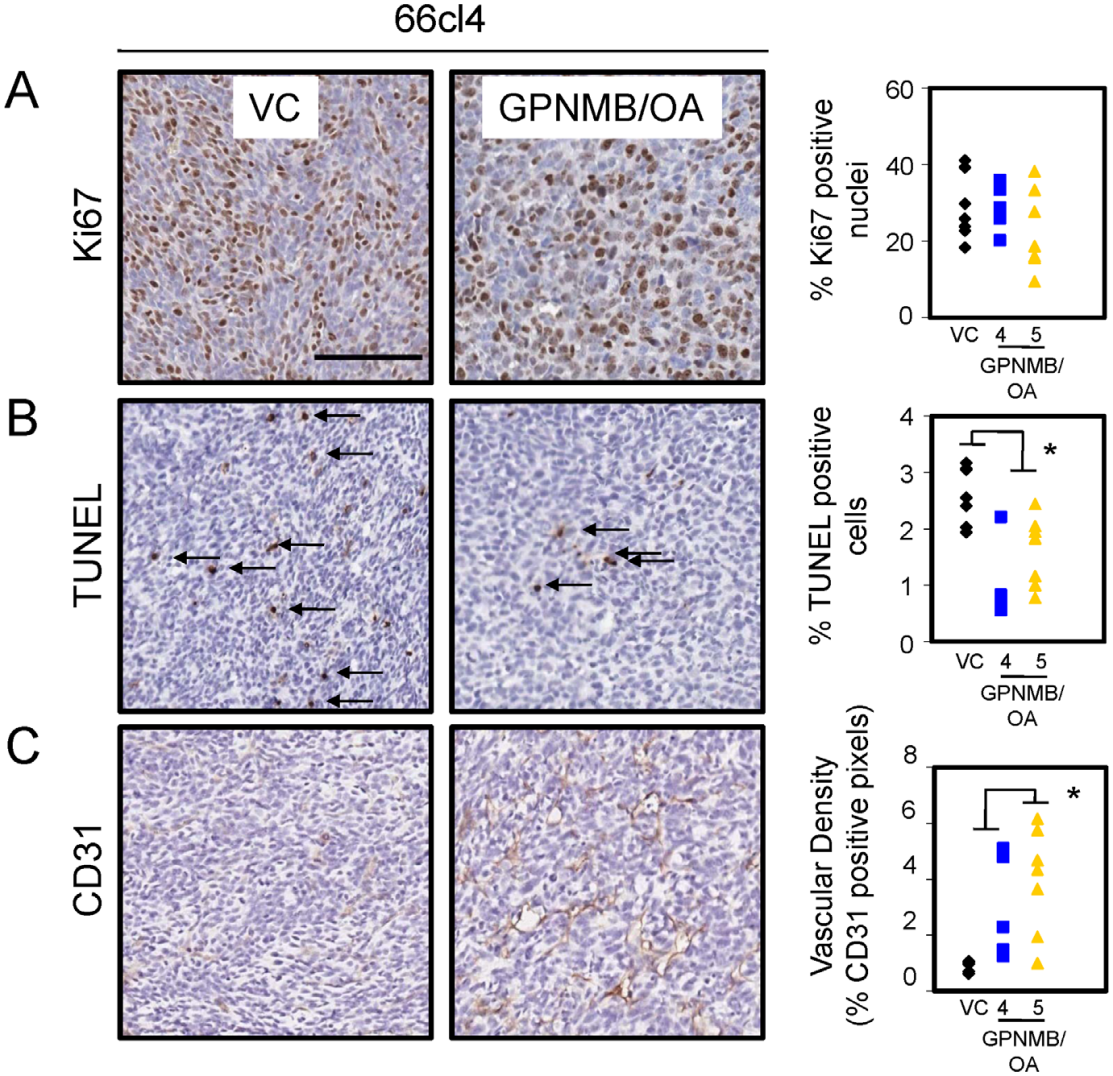
Osteoactivin inhibits apoptosis and enhances angiogenesis in 66cl4-derived mammary tumors. Tumors derived from vector control or Osteoactivin-expressing 66cl4 cells were characterized using immunohistochemical analysis for (**A**) proliferation (Ki67), (**B**) apoptosis (TUNEL) and (**C**) vascular density (CD31). Representative images are shown for control tumors (VC) or GPNMB/OA5-expressing tumors (left panels). Proliferation and apoptosis are expressed as the percentage of Ki67 or TUNEL-positive nuclei/field, respectively. Vascular density is expressed as the percentage of total CD31-positive pixels/field.

To better characterize the functional role of GPNMB/OA in promoting tumor growth, we removed the primary tumors and subjected them to IHC analysis to assess differences in proliferation, apoptosis and angiogenesis. Using antibodies against Ki67 as a proliferation marker [26], we observed no significant differences in the mean percentage of proliferation control (28.7%) versus GPNMB/OA-expressing mammary tumors (25.1%) (**Figure 2A**). We next quantified the number of apoptotic cells in non-necrotic regions of these mammary tumors and found that, on average, fewer cells in GPNMB/OA-expressing tumors (1.1%) were undergoing apoptosis when compared to control mammary tumors (2.6%) (**Figure 2B**). Finally, we assessed the vascular density of these tumors by quantifying the degree of CD31 positivity, a routinely used endothelial cell marker. These analyses revealed that the vascular density in GPNMB/OA-expressing mammary tumors (3.5%) was significantly higher when compared to control tumors (0.9%) (**Figure 2C**). We next investigated whether this increase in angiogenesis could be attributed to VEGF induction by GPNMB/OA. Similar quantities of VEGF were detected in cell lysates and conditioned media from vector control and GPNMB/OA expressing 66cl4 cells (**Supplemental Figure S2 A, B**); however, tumors derived from GPNMB/OA expressing cells (mean 652ng/ml) produced nearly twice as much VEGF as vector control tumors (mean = 328 ng/mL), suggesting that GPNMB/OA may indirectly upregulate VEGF *in vivo* via interactions with stromal cells in the tumor microenvironment (**Supplemental Figure S2C**).

To address whether the GPNMB/OA-associated angiogenic phenotype was specific to the 66cl4 mouse mammary tumor model, we next interrogated the association between GPNMB/OA expression and vascular density in human breast cancer cells and primary tumors. We ectopically expressed GPNMB/OA in BT549 cells, a basal breast cancer model. Although vector control and GPNMB/OA-expressing BT549 cells were incapable of forming tumors when injected into athymic mice (data not shown), we analyzed whether GPNMB/OA is capable of enhancing the angiogenic phenotype of these cells by performing matrigel plug assays. Matrigel plugs containing either vector control or GPNMB/OA-expressing BT549 cells were harvested 10 days post-injection and subjected to immunohistocytochemical analysis for CD31 expression. These analyses, in agreement with our results from GPNMB/OA-expressing 66cl4 mouse mammary tumors, revealed that matrigel plugs containing GPNMB/OA-expressing BT549 cells displayed greater endothelial recruitment (11.8%) when compared to matrigel plugs composed of empty vector control cells (8.5%) (**Supplemental Figure S3A, B**).

**Figure 3:**
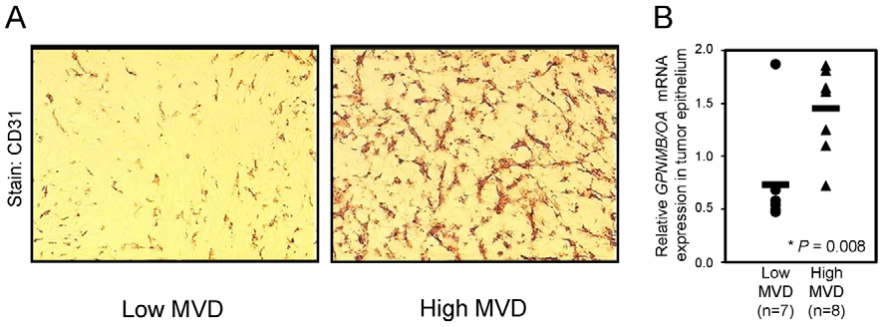
GPNMB/OA-expressing human mammary tumors display enhanced vascular density. (**A**) Human breast tumors were stained with CD31 and classified into two groups, those with low or high microvascular density (MVD). (**B**) Laser capture microdissection was used to extract RNA specifically from the tumor epithelium of low or high MVD breast tumors. Analysis of *GPNMB/OA* mRNA expression values for each tumor revealed a significant correlation between high levels of GPNMB/OA expression in the tumor epithelium and high microvascular density. *, P = 0.008, Student's t-test.

We next interrogated gene expression data from laser capture microdissected tumor epithelium isolated from breast tumors that were categorized as high versus low MVD, based on quantification of CD31 staining [27] (**Figure 3A**). Interestingly, we observed a 2-fold increase in *GPNMB/OA* mRNA levels in the epithelium of breast tumors characterized as high MVD (average expression value = 1.452) versus those with low MVD (average expression value = 0.734) (**Figure 3B**). These data, together with our observations from our mouse and human breast cancer models, suggest a role for GPNMB/OA in promoting endothelial recruitment during mammary tumorigenesis.

### GPNMB/OA extracellular domain is shed by ADAM10

**Figure 4:**
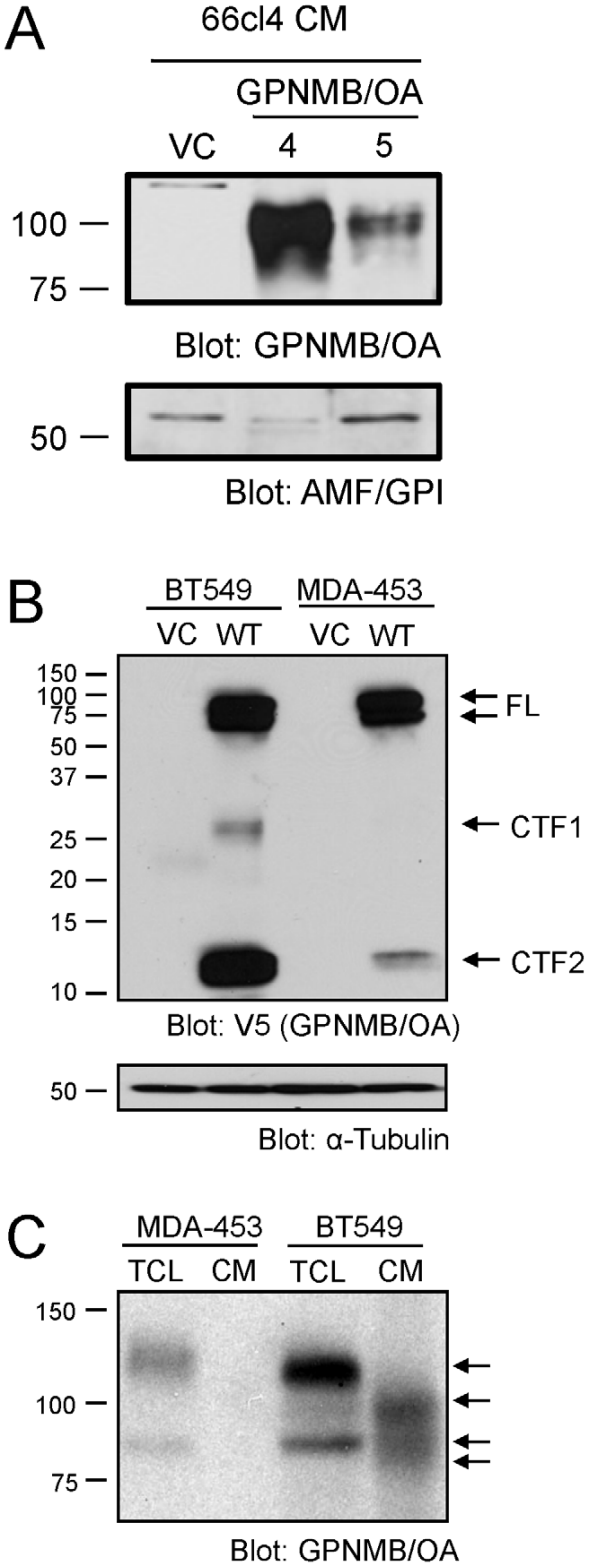
The GPNMB/OA ectodomain is shed from breast cancer cells. (**A**) The shed form of GPNMB/OA was detected in the conditioned media from 66cl4 cells engineered to overexpress this protein. AMF/GPI is a secreted cytokine that served as a loading control for the conditioned media collected from VC, GPNMB/OA4 and GPNMB/OA5-expressing cells (**B**) Anti-V5 immunoblot identified full length GPNMB/OA as well two C-terminal fragments (CTF1 and CTF2) in human breast cancer cells engineered to overexpress GPNMB/OA (BT549-WT and MDA-MB-453-WT). Breast cancer cells harboring an empty vector (VC) served as negative controls. Immunoblots for α-Tubulin were performed to control for protein loading in whole cell lysates. (**C**) Immunoblot analysis with an antibody directed to the extracellular domain of GPNMB/OA identified shed GPNMB/OA in the conditioned media (CM) harvested from GPNMB/OA-expressing BT549 cells (WCL: whole cell lysate).

It has been previously reported that GPNMB/OA can be cleaved and shed from the cell surface, producing an ECD fragment that has signaling capacity in stromal cells [20], [21]. The GPNMB/OA ECD, when fused to the immunoglobulin Fc region, is also capable of binding to the surface of endothelial cells [28]. Given these observations, we hypothesized that the shed, soluble fragment of GPNMB/OA might facilitate the GPNMB/OA-dependent pro-angiogenic phenotype. To investigate this hypothesis, we determined whether the GPNMB/OA ECD was consistently shed into the conditioned media of breast cancer cells. Indeed, we can detect a soluble form of GPNMB/OA in conditioned media from GPNMB/OA-expressing 66cl4 cells (**Figure 4A**). To extend these observations to a human breast cancer model, we engineered two cell lines overexpressing GPNMB/OA. Full length GPNMB/OA, containing a C-terminal V5-epitope tag, was readily detectable in the basal-like BT549 breast cancer cells and luminal-like MDA-MB-453 cells engineered to overexpress this protein (**Figure 4B**). In addition to full length GPNMB/OA, we also identified two small C-terminal fragments with molecular weights of approximately ∼25kDa and ∼13kDa, which we labeled CTF1 and CTF2, respectively (**Figure 4B**). These fragments are products of post-translational proteolytic processing and have previously been described in C2C12 myoblast cells engineered to overexpress GPNMB/OA, as well as in melanocytes and melanoma cells endogenously expressing GPNMB/OA [9], [20]. Notably, processing of GPNMB/OA was less efficient in MDA-MB-453 cells relative to that observed in BT549 cells, despite comparable expression levels of the full length protein in both cell lines (**Figure 4B**). In addition, we observed that less GPNMB/OA ECD was shed into conditioned media of MDA-MB-453 cells when compared to conditioned media harvested from BT549 cells (**Figure 4C**).

**Figure 5:**
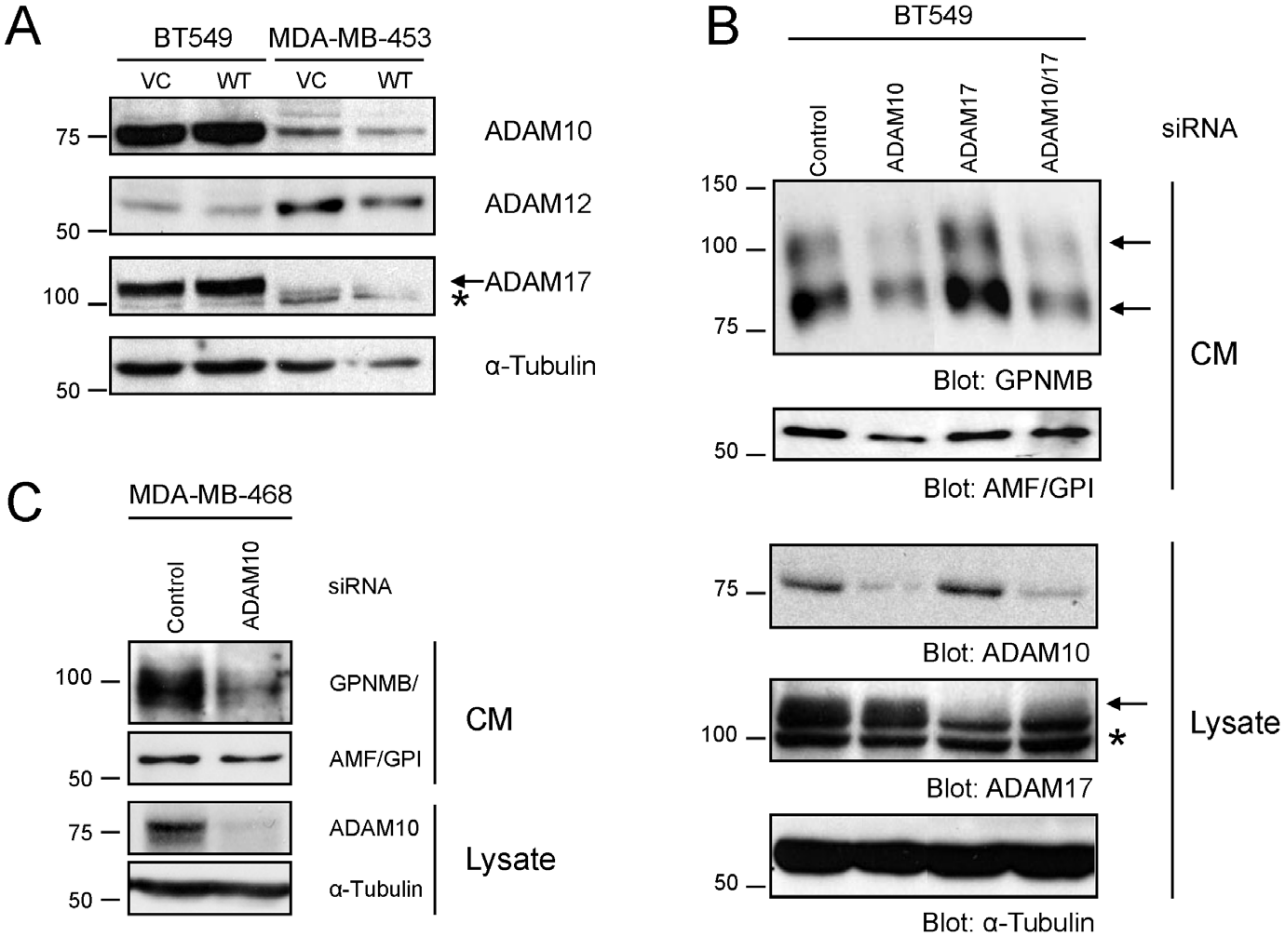
ADAM10 induces shedding of the GPNMB/OA ectodomain. (**A**) Immunoblot analysis of ADAM10, ADAM12 and ADAM17 expression in BT549 and MDA-MB-453 cells. Arrow indicates band corresponding to Adam17 and asterisk denotes a doublet of non-specific bands. (**B**) siRNA-mediated knockdown of ADAM10, but not ADAM17, reduced shedding of GPNMB/OA in BT549 cells. *Upper panels*, immunoblot analysis for GPNMB/OA in the CM harvested from BT549-GPNMB/OA cells treated with the indicated control and ADAM-specific siRNAs. *Lower panels*, immunoblot analysis was performed to determine the degree of ADAM10 and ADAM17 knockdown. Arrow indicates band corresponding to Adam17 and asterisk denotes a doublet of non-specific bands. (**C**) A role for ADAM10 in GPNMB/OA ectodomain shedding is confirmed in MDA-MB-468 human breast cancer cells that endogenously express GPNMB/OA. An immunoblot for GPNMB/OA was performed on CM harvested from MDA-MB-468 breast cancer cells treated with control or ADAM10-specific siRNAs. Immunoblot analysis with antibodies specific for ADAM10 was performed to confirm knockdown of ADAM10 expression. Immunoblots for α-Tubulin were performed to control for protein loading in whole cell lysates (**A**, **B**, and **C**). Immunoblots for AMF/GPI were performed to control for protein loading in the CM samples (**B**, **C**). CM refers to conditioned media, Lysate indicates whole cell lysates prepared from these cells.

The mechanism governing GPNMB/OA shedding has been the subject of growing interest, yet the specific proteases involved in this process have yet to be elucidated. The ADAM (A Disintegrin And Metalloproteinase) subfamily of matrix metalloproteinases (MMPs), known for their sheddase abilities, have been recently postulated to be candidate proteases that could mediate GPNMB/OA ectodomain shedding [9]. To test this possibility, we first investigated whether ADAM10, 12 or 17 were differentially expressed between BT549 and MDA-MB-453 cells, which differ in their degree of GPNMB/OA shedding. We found that both ADAM10 and ADAM17 were expressed at higher levels in BT549 cells compared to MDA-MB-453 cells, whereas ADAM12 expression was higher in MDA-MB-453 cells (**Figure 5A**). We next asked whether ADAM10 or ADAM17 - which are the primary sheddases for most ectodomains [29] - were functionally required for GPNMB/OA shedding. To accomplish this, we performed transient siRNA mediated knockdown of ADAM10 and ADAM17, independently or in combination, in GPNMB/OA-expressing BT549 cells and found that the amount of the GPNMB/OA ECD detectable in the conditioned media was diminished only when ADAM10 expression was reduced (**Figure 5B** **,** ***upper panels***). Moreover, co-suppression of ADAM17 and ADAM10 did not further diminish release of the soluble GPNMB/OA ECD. Immunoblots for GPI were performed to control for the total amount of protein in the condition media (**Figure 5B** **,** ***upper panels***). Immunoblots for ADAM10 and ADAM17 revealed that the siRNA-mediated knock-down of these proteins was effective (**Figure 5B** **,** ***lower panels***). To confirm whether ADAM10 was required for GPNMB/OA shedding in an independent cell line, we chose the basal-like MDA-MB-468 cell line that endogenously expresses GPNMB/OA. Indeed, transient siRNA-mediated knockdown of ADAM10 in these cells also greatly diminished shedding of the GPNMB/OA ECD into the culture media (**Figure 5C**). Together, these data indicate that ADAM10 is able to release the GPNMB/OA ectodomain from the surface of breast cancer cells.

### GPNMB/OA ECD promotes endothelial cell migration

**Figure 6:**
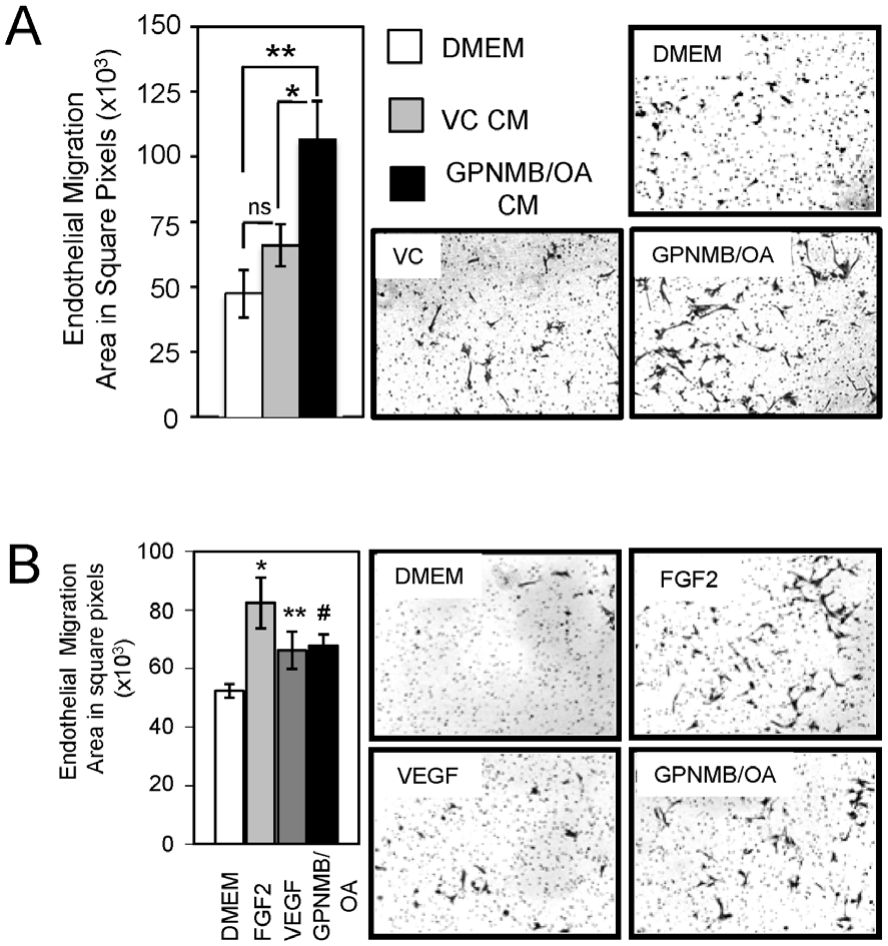
GPNMB/OA ECD promotes endothelial migration. (**A**) Human pulmonary microvascular endothelial cells (HPMECs) were plated onto the upper well and allowed to migrate towards serum free media (DMEM) or conditioned media (CM) harvested from vector control (VC) or GPNMB/OA-expressing cells (GPNMB/OA). The area in square pixels was quantified over fifteen images for each condition (*left panel*), one representative field for each condition is shown (*right panel*). The data is the average of three independent experiments performed in triplicate and the standard error is shown: *; P<0.006; **; P<0.0007. (**B**) HPMECs were plated in the upper chamber and allowed to migrate towards serum free media containing recombinant FGF2 (50 ng/ml), VEGF (50 ng/ml) or GPNMB/OA (rhECD, 100ng/ml). Quantification (*left panel*) was performed as described in (**A**) and one representative field for each condition is shown (*right panel*). The data is the average of three independent experiments performed in triplicate. The standard error is shown. All P-values were determined by t-test, comparing the experimental condition to untreated control: *, P = 0.0014; **, P = 0.0138; #, P = 0.0005).

Having determined that GPNMB/OA is constitutively shed in an ADAM10-dependent manner in our breast cancer model systems, we next investigated whether this shed GPNMB/OA ECD possessed angiogenic properties. Given that the migration of endothelial cells is a requisite step during tumor angiogenesis, we investigated whether the GPNMB/OA ECD was capable of promoting this process. First, we collected conditioned media (CM) from empty vector control (VC) or GPNMB/OA-expressing BT549 cells and used this as chemoattractant for HPMEC endothelial cells *in vitro*. We found that CM from VC cells induced limited endothelial migration; however, this increase did not achieve statistical significance when compared to the effects of serum free media (DMEM) (**Figure 6A**). In contrast, CM from GPNMB/OA-expressing BT549 cells induced a >2-fold enhancement in endothelial migration when compared to serum free media (**Figure 6A**). To determine whether this effect on endothelial migration was specific to shed GPNMB/OA, we used a V5-tagged recombinant protein encoding only the ECD of GPNMB/OA (rhECD). In this assay, known inducers of endothelial migration such as FGF-2 and VEGF, promoted a >2 fold or 1.5 fold increase in endothelial migration, respectively, when compared to serum free media (DMEM) (**Figure 6B**). By comparison, we found that recombinant human GPNMB/OA ECD induced a 1.5-fold increase in endothelial migration compared to serum free media (**Figure 6B**). Together, these observations support the hypothesis that shed GPNMB/OA augments breast tumor angiogenesis by directly stimulating endothelial migration.

## Discussion

We have previously demonstrated that GPNMB/OA expression is elevated during the formation of primary mammary tumors; its expression is further elevated in breast cancer bone metastases and plays a functional role in this process [18]. GPNMB/OA belongs to a group of osteomimetic proteins (ie. Osteopontin, Osteonectin and Osteocalcin) [30] that are normally expressed by osteoblasts/osteoclasts, which when expressed in cancer cells, promote the development of bone metastases. Indeed, GPNMB/OA is emerging as a critical mediator of osteoblast and osteoclast differentiation, two cell types important for bone remodeling and turnover [2], [3], [4]. In addition, GPNMB/OA expression is up-regulated in bone pathologies such as osteoarthritis and during fracture repair [12], [31]. However, in the current study, we demonstrate that GPNMB/OA expression is also elevated in 4T1 subpopulations that preferentially metastasize to lung and liver, in addition to those that spread to bone. This data suggests that the GPNMB/OA may play a more generalized role in promoting tumor progression, but does not preclude the possibility that certain GPNMB/OA-related functions specifically favor the development of bone metastases.

We have observed that in certain cell-based models, such as 66cl4 mouse mammary carcinoma cells, GPNMB/OA expression can enhance tumor growth *in vivo*. Our data suggests that GPNMB/OA-dependent augmentation of tumor growth is attributed to decreased apoptosis and increased angiogenesis in GPNMB/OA expressing tumors. It is not clear whether the predominant tumor growth stimulatory effect of GPNMB/OA stems from impaired apoptosis or enhanced vascular recruitment; however, it is likely that the two processes are interrelated. Indeed, breast cancer cells that overexpress GPNMB/OA, when grown in complete media, tend to display slower *in vitro* growth rates when compared to empty vector control cells [19], suggesting that the reduced apoptosis observed in GPNMB/OA-expressing mammary tumors may be secondary to tumor/stromal interactions that occur only *in vivo*. Recently, an alternate mechanism, involving GPNMB/OA-mediated suppression of T-cell activation, has been proposed to explain how GPNMB/OA can promote the growth of melanoma tumors [25]. In this study, shRNA mediated reduction in GPNMB/OA expression in B16 melanoma cells was shown to cause a reduction in sub-cutaneous tumor growth compared to control cells when injected into syngeneic mice. Interestingly, this difference in melanoma growth between GPNMB/OA-expressing cells and those with the GPNMB/OA knockdown was not observed when these cells were injected into immunodeficient mice [25]. The mechanism by which GPNMB/OA promoted melanoma tumor outgrowth was through suppression of T-cell activation, which normally serves to limit tumor outgrowth [25]. Given that we employed a syngeneic mouse breast cancer model to initiate our studies, we examined this possibility and found that GPNMB/OA expression was able to promote the growth of 66cl4 cells in both an immunocompetent and immunocompromised background. These observations indicate that GPNMB/OA can contribute to tumor growth through mechanisms other than suppression of anti-tumor immunity.

The observation that primary human breast tumors with high MVD express elevated levels of GPNMB/OA in the tumor epithelium provides a clinical correlate that substantiates our *in vivo* studies with the 66cl4 mammary carcinoma model. Importantly, we restricted these analysis to examining GPNMB/OA expression in the tumor epithelium of high and low MVD primary breast tumors; therefore, it remains to be determined whether GPNMB/OA expression in the tumor stroma is also associated with enhanced angiogenesis. Of interest is the observation that tumor–derived endothelial cells express high levels of GPNMB/OA relative to endothelial cells derived from normal tissues [32]. However, it is unclear whether GPNMB/OA expressed within endothelial cells functions to promote angiogenesis. Our data suggests that GPNMB/OA, when expressed in breast cancer cells, can increase vascular recruitment and enhance tumor growth.

Interestingly, VEGF levels in GPNMB/OA expressing 66cl4 cells is similar to empty vector control cells when measured *in vitro*; however, VEGF expression is upregulated ∼2-fold in GPNMB/OA-expressing compared to VC mammary tumors. Given that GPNMB/OA is only capable of inducing VEGF expression *in vivo*, it is likely that GPNMB/OA promotes interactions with and/or recruitment of stromal cells, which in turn produce increased amounts of VEGF. Potential stromal cell types that could be involved in this process are tumor-associated macrophages (TAMs). These cells are actively recruited into breast tumors and are known to produce VEGF, which contributes to angiogenesis and breast tumor growth [33]. Whether GPNMB/OA-expressing mammary tumors are characterized by increased numbers of infiltrating TAMs requires further investigation.

In addition to its ability to indirectly upregulate VEGF *in vivo*, we investigated whether GPNMB/OA may be able to promote angiogenesis via direct interactions with endothelial cells. Recent studies demonstrating that GPNMB/OA can undergo proteolytic processing led us to investigate the possibility that this protein was subject to ectodomain shedding in breast cancer cells. We are the first to identify ADAM10 as specific protease capable of cleaving and releasing the ECD of GPNMB/OA. This observation is consistent with published reports showing that GPNMB/OA shedding can be inhibited by GM6001, a broad spectrum MMP-inhibitor [9], [20], [21]. GPNMB/OA processing can also be induced through the use of a calmodulin inhibitor or via PMA stimulation [9]. It has been proposed that ADAM10 can promote the constitutive shedding of target proteins, such as CD44, whereas PMA-induced CD44 shedding is mediated through ADAM17 [34]. In our study, we specifically investigated whether ADAM10 and ADAM17 were responsible for constitutive shedding of GPNMB/OA in breast cancer cells, thus it is possible that ADAM17 is also capable of shedding GPNMB/OA in the context of PMA stimulation.

Our data indicate that the soluble extracellular domain of GPNMB/OA can function as a chemoattractant for endothelial cells, which is capable of inducing the migration of this cell type. While the receptor for the GPNMB/OA ECD in endothelial cells is not known, the literature implicates a number of interesting candidates. For example, GPNMB/OA can be immunoprecipitated with either integrin β1 or integrin β3 in differentiating osteoclasts [2]. Presumably this interaction occurs via the N-terminal RGD domain in GPNMB/OA, which is functionally required for its ability to adhere to endothelial cells [2]. An increasing body of evidence supports a role for the β1 class of integrins in regulating endothelial adhesion, migration and survival during tumor induced angiogenesis [35]. The β3 integrin, as part of the αVβ3 receptor, is expressed on the surface of endothelial cells during angiogenesis and has been reported to interact with and potentiate FGF-2 signaling in endothelial cells [36]. Thus, integrins may serve as receptors for the GPNMB/OA ECD and transduce signals that promote endothelial migration.

The effects of GPNMB/OA rhECD on endothelial migration are significant but more modest than the effects of CM containing shed GPNMB/OA ECD, suggesting that GPNMB/OA cooperates with other factors to promote endothelial migration and angiogenesis. It is conceivable that the shed form of GPNMB/OA can act directly to induce endothelial migration, which in concert with an indirect upregulation of VEGF, leads to a robust angiogenic response.

Given the growing interest in GPNMB/OA targeted agents in breast cancer [22], [23], our observations that ADAM10 functions as a sheddase for GPNMB/OA have potentially important therapeutic implications. CDX-011 is an anti-GPNMB/OA antibody-drug conjugate whose efficacy is proportional to the levels of cell surface GPNMB/OA expressed on cancer cells [19], [21]. Thus, GPNMB/OA shedding from the cell surface may limit the efficacy of GPNMB/OA-targeted therapies. It is possible that agents such as CDX-011 might be improved when used in combination with ADAM10 inhibitors that would reduce ECD shedding of GPNMB/OA. One such inhibitor, INCB7839, has been shown to cooperate with receptor tyrosine kinase inhibitors that target EGFR and ErbB2 to impair breast tumor growth [37]. Similarly, epirubicin, a chemotherapeutic drug known to down-regulate ADAM10 expression in cancer cells [38], when used in combination with CDX-011, could potentially enhance its efficacy.

## Materials and Methods

### Ethics Statement

Studies involving laser capture microdissection of human breast tumor samples and subsequent gene expression analysis were approved by the McGill University Health Centre Research Ethics Board (Protocols SUR-99-780 and SUR-00-966). All patients provided written, informed consent.

For experiments requiring animal use, the mice were housed in facilities managed by the McGill University Animal Resources Centre and all animal experiments were conducted under a McGill University approved Animal Use Protocol (#4830) in accordance with guidelines established by the Canadian Council on Animal Care.

### Cell culture and transfections

The murine 4T1 and human BT549, MDA-MB-453 and MDA-MB-468 breast cancer cell lines used in this study were obtained from the ATCC and cultured according to their guidelines. The 66cl4 murine mammary carcinoma cells were a generous gift from Dr. Fred Miller (Barbara Ann Karmanos Cancer Institute, Detroit, MI). All 4T1-derived subpopulations were generated by *in vivo* selection in our lab [18; Tabariès and Siegel, unpublished data]. Human pulmonary microvascular endothelial cells (HPMEC-ST1-6R) have been described previously [39] and were a generous gift from Dr. Vera Krump-Konvalinkova (IPEK-LMU, Munich, Germany). The pEF1-GPNMB/OA vector was constructed by ligating the full-length human GPNMB/OA cDNA (Open Biosystems; Accession: BC032783) into a pEF1/V5-His expression vector (Invitrogen) using 5′ *Eco* RI and 3′ *Not* I restriction enzyme sites. BT549 and MDA-MB-453 cell lines were engineered to express GPNMB/OA by LipofectAMINE 2000 (Invitrogen)–mediated transfection. GPNMB/OA-expressing BT549 cells are pools of 3 independent clones. Osteoactivin-expressing 66cl4 cells have been described previously [18]. Transient knockdown of ADAM10 and ADAM17 was accomplished by transfection (Lipofectamine 2000, Invitrogen) using 15 nM of the ON-TARGETplus SMARTpool [pool of four ADAM10 or ADAM17-targeted small interfering RNAs (siRNA), Dharmacon]. An ON-TARGETplus pool of four non-targeting (scrambled) siRNAs was used as a control. Twenty-four hours later the transfection media was removed, the cells were washed once with PBS and media was changed to serum free media (SFM). Conditioned media, used for immunoblot or ELISA analysis, was collected after 48 hours.

### Immunoblotting

Sub-confluent cells were lysed for 20 min. on ice in TNE lysis buffer. Protein concentrations were determined by Bradford assay (Bio-Rad) and 30–45 µg of total protein were used in gel electrophoresis. For immunoblotting of conditioned media (CM), 1mL of CM was concentrated using microcentricon tubes (30kDa MWCO, Millipore) and 10uL of protein the concentrate was loaded on a gel. The antibodies used were as follows: GPNMB/OA (1∶2,500 dilution; R&D Systems), Osteoactivin (1∶2,500 dilution; R&D Systems), ADAM10 (1∶1,000 dilution; Millipore), ADAM17 (1∶1,000 dilution; Millipore), ADAM12 (1∶200 dilution; rb122) [40], GPI (1∶1,000 dilution; Santa Cruz), V5 (1∶5,000 dilution, Sigma) and α-Tubulin (1∶10,000 dilution; Sigma-Aldrich). Appropriate horseradish peroxidase–conjugated secondary antibodies (Jackson ImmunoResearch Laboratories) were used at a dilution of 1∶10,000 and proteins were visualized by chemiluminescence (Millipore).

### In vivo tumor growth assays

Female Balb/c mice (4–6 weeks) were purchased from Charles River Laboratories (Wilmington, MA). For the tumor growth assays, 66cl4 mammary carcinoma cells were harvested from sub-confluent plates, washed once with PBS and resuspended (10^4^ cells) in 50µl of a 50∶50 solution of matrigel (BD Biosciences) and PBS. This cell suspension was injected into the right abdominal mammary fat pad of Balb/c mice and measurements were taken beginning on day 10 post injection for the time periods indicated. Tumor volumes were calculated using the following formula: π*LW* ^2^/6, where *L* is the length and *W* is the width of the tumor. Tumors were surgically removed, using a cautery unit, once they reached a volume between 200–300 mm^3^.

### Matrigel plug assays

Female athymic mice (4–6 weeks) were purchased from Charles River Laboratories (Wilmington, MA). Subconfluent BT549 cells were trypsinized, washed once in PBS and resuspended at a final concentration of 1×10^7^ cells/mL in matrigel. A 100µL cell suspension was injected subcutaneously into athymic mice. Mice were sacrificed and matrigel plugs were removed 10 days post-injection. The matrigel plugs were then fixed overnight in 4% paraformaldehyde and prepared for immunohistocytochemical staining.

### Immunohistocytochemical staining and analysis of 66cl4 mammary tumors

Primary mammary tumors were fixed overnight in 4% paraformaldehyde. Immunohisto-cytochemistry was performed with the following antibodies: Ki67 (1∶100 dilution; BD Pharmingen; Mississauga, ON) and CD31 (1∶200 dilution; BD Pharmingen). Appropriate Biotin-SP-conjugated anti-IgG secondary antibodies were purchased from Jackson Laboratories (Bar Harbor, ME). Apoptotic cells were detected using an ApopTag® Peroxidase *In Situ* Apoptosis Detection Kit (Chemicon® International; Temecula, CA) in accordance with the manufacturer's instructions. Sections were developed with 3-3-diaminobenzidine-tetrahydrochloride and counterstained with hematoxylin. Slides were first scanned using a Scanscope XT digital slide scanner (Aperio, Vista, CA, USA) and further analyzed with Imagescope software (Aperio) using either positive pixel count or immunohistocytochemistry nuclear algorithms. For Ki67 and TUNEL staining, data was represented as a percentage of positive nuclei among total nuclei in each field. For CD31 analyses only moderate (+2) and strong (+3) staining were considered positive. The number of positive pixels is represented as a percentage of total pixels per field (66cl4 tumors) or as CD31-positive pixels per nuclei (BT549 plugs).

### Assessment of GPNMB/OA expression and MVD in human breast tumors

Immunohistocytochemistry directed against PECAM1 (Cat. No. BBA7, R&D Systems, Minneapolis, MN) was performed for MVD quantitation according to antibody manufacturer's instructions. Quantitation of PECAM1 staining density was performed by averaging the stained pixel intensity of 3 fields captured at 10× magnification using imageJ (http://rsb.info.nih.gov/ij/). Tissue samples from 21 patients undergoing surgery for primary invasive ductal carcinoma (IDC) with no prior neoadjuvant therapy were subjected to Laser Capture Microdissection (LCM); these were selected from more than 100 tumors based on their having the highest and lowest MVD. From this cohort we obtained 17 samples of tumor-associated vasculature. LCM, RNA isolation and sample preparation, as well as microarray hybridization, were carried out as previously described [27], [41]. Microarray data were extracted and analyzed as previously described [27]. Normalized GPNMB/OA expression values were determined from the following Agilent probe: A_23_P134426. To minimize the effect of outliers in this small subset of tumors, we removed the tumors with highest and lowest GPNMB/OA expression from both groups (high MVD and low MVD). This resulted in the following sample sizes low MVD (n = 7) and high MVD (n = 8). Student's T-test (2 tails) was used to assess statistical significance.

### Quantification of shed GPNMB/OA and soluble VEGF

The GPNMB/OA ELISA assay was designed by coating 96-well plates with capture antibody (human anti-GPNMB/OA, 2.10.2), which was generously provided by Celldex Therapeutics, at 4°C overnight. After blocking with BSA and several wash steps, conditioned media from BT549 cells was allowed to adhere to the antibody-coated plate for one hour at 37°C. A goat anti-GPNMB/OA antibody (R&D systems, Minneapolis, MN) was used as a detection antibody and an HRP-conjugated donkey anti-goat antibody (Jackson ImmunoResearch Laboratories) in concert with TMB (3,3′,5,5′-tetramethylbenzidine) chromogenic substrate (Pierce Thermoscientific, Rockford, IL) was used to visualize the reaction. Plates were quantified with 540/450 nm wavelength readings on a Bio-Plex Reader (Bio-Rad Laboratories, Hercules, CA) and data was analyzed with Bio-Plex Manager 2.0 software (Bio-Rad Laboratories, Hercules, CA). Soluble VEGF was quantified using manufacturer's protocol with a commercially available ELISA kit (R&D systems, Minneapolis, MN).

### Endothelial migration assays

For assessing endothelial migration, a GPNMB-specific ELISA was used to quantify the amount of GPNMB/OA sECD present in the CM of GPNMB/OA-expressing BT549 cells that had been cultured for two days in serum free media. A volume of media containing 50ng of the sECD was added to a final volume of 1 mL of serum-free media and placed in the bottom chamber of a modified Boyden chamber assay. The same volume of concentrated conditioned media was added from the vector control cells. Untreated refers to serum free media (SFM) that was not mixed with conditioned media harvested from GPNMB/OA-expressing or vector control cells. Briefly, 7.5×10^4^ HPMECs were seeded in the upper well and allowed to migrate through an 8µm porous membrane towards the conditioned media in the bottom chamber for a period of 18 hours. For EC migration experiments employing recombinant factors, recombinant FGF2 (50 ng/ml; BPS Bioscience, San Diego, CA), VEGF (50 ng/ml; BPS Bioscience, San Diego, CA) and GPNMB/OA (rhECD, 100ng/ml; Celldex, Needham, MA) was added to 1mL SFM in the bottom chamber, and 1×10^5^ HPMECs were plated in the upper chamber and allowed to migrate through the filter for a period of 18 hours. At the termination of each experiment, cells were fixed in formalin, stained with crystal violet (Sigma) and those cells remaining on the upper side of the membrane were removed by scraping. Five images were taken for each insert and the cells were quantified using Scion Image software (Scion Corporation). Data for each insert are represented as the average pixel count from the five images. The data was obtained from at least three independent experiments, performed in triplicate wells.

## Supporting Information

Figure S1 Tumors derived from a pool of GPNMB/OA expressing 66cl4 cells display enhanced tumor outgrowth in immunocompetent Balb/c and athymic mice. (A) GPNMB/OA expression was confirmed by immunoblot analysis of total cell lysates from pooled vector control (VC) and GPNMB/OA-expressing (GPNMB/OA pool) 66cl4 cells. As a loading control, total cell lysates were blotted for α-Tubulin. (B) Tumor growth curves from Balb/c (triangles) and athymic (circles) mice injected with 1×105 VC (open symbols) or GPNMB/OA pool (filled symbols) expressing 66cl4 cells. *, P = 0.0003, GPNMB/OA pool (athymic) vs. VC (athymic); **, P<0.0001, GPNMB/OA pool (Balb/c) vs. VC (Balb/c); #, P = 0.0001, GPNMB/OA (Balb/c) vs. GPNMB/OA (athymic). All P-values were determined using a non-parametric Mann-Whitney test for serial measurements.
(0.65 MB TIF)

Figure S2 Analysis of VEGF expression and endothelial recruitment in breast cancer cells expressing GPNMB/OA. (A) Total cell lysates and (B) cell supernatants were extracted from vector control (VC, black bars) and GPNMB/OA-expressing (GPNMB/OA4, blue bars) 66cl4 cells grown in vitro and from (C) tumors grown in vivo. Tumors were excised at a volume of 200–300mm3 and flash frozen in liquid nitrogen. VEGF protein was quantified using ELISA and normalized to the total amount of protein in the corresponding cell lysate (A, B) or tumor lysates (C). *, P = 0.003, Students t-test.
(0.52 MB TIF)

Figure S3 GPNMB/OA promotes angiogenesis in an in vivo human breast cancer model. VC or GPNMB/OA-expressing BT549 cells (1×106) were suspended in a 50∶50 solution of PBS∶matrigel and injected subcutaneously into athymic mice and the animals sacrificed 10 days later. (A) CD31 (endothelial marker)-stained pixels were quantified for each matrigel plug and normalized to the number of total nuclei in the section. *, P = 0.021, Students t-test. (B) Vasculature recruited into the matrigel plugs was visualized on the inner surface of the skin (upper panels). Representative images of CD31 stains are shown (lower panels). Scale bars represent 100 µm.
(1.96 MB TIF)
