# Supplementary figures and images for: ADAM10 Releases a Soluble Form of the GPNMB/Osteoactivin Extracellular Domain with Angiogenic Properties
Source: PLoS One. 2010 Aug 10;5(8):e12093. doi: 10.1371/journal.pone.0012093 (PMC2919417; doi:10.1371/journal.pone.0012093)

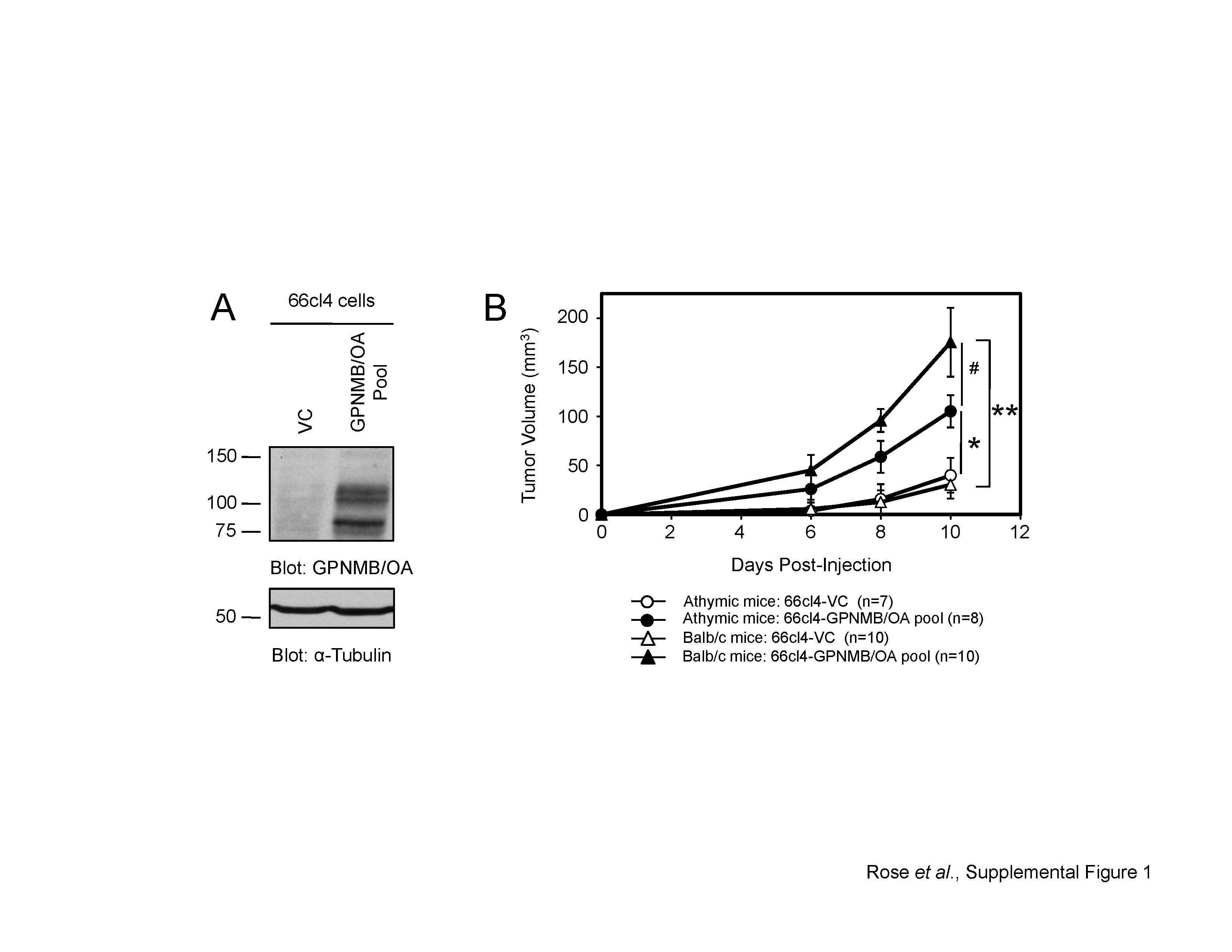

Supplement: Figure S1 — Tumors derived from a pool of GPNMB/OA expressing 66cl4 cells display enhanced tumor outgrowth in immunocompetent Balb/c and athymic mice. (A) GPNMB/OA expression was confirmed by immunoblot analysis of total cell lysates from pooled vector control (VC) and GPNMB/OA-expressing (GPNMB/OA pool) 66cl4 cells. As a loading control, total cell lysates were blotted for α-Tubulin. (B) Tumor growth curves from Balb/c (triangles) and athymic (circles) mice injected with 1×10^5^ VC (open symbols) or GPNMB/OA pool (filled symbols) expressing 66cl4 cells. *, P = 0.0003, GPNMB/OA pool (athymic) vs. VC (athymic); **, P<0.0001, GPNMB/OA pool (Balb/c) vs. VC (Balb/c); #, P = 0.0001, GPNMB/OA (Balb/c) vs. GPNMB/OA (athymic). All P-values were determined using a non-parametric Mann-Whitney test for serial measurements. (0.65 MB TIF) [file pone.0012093.s001.tif]

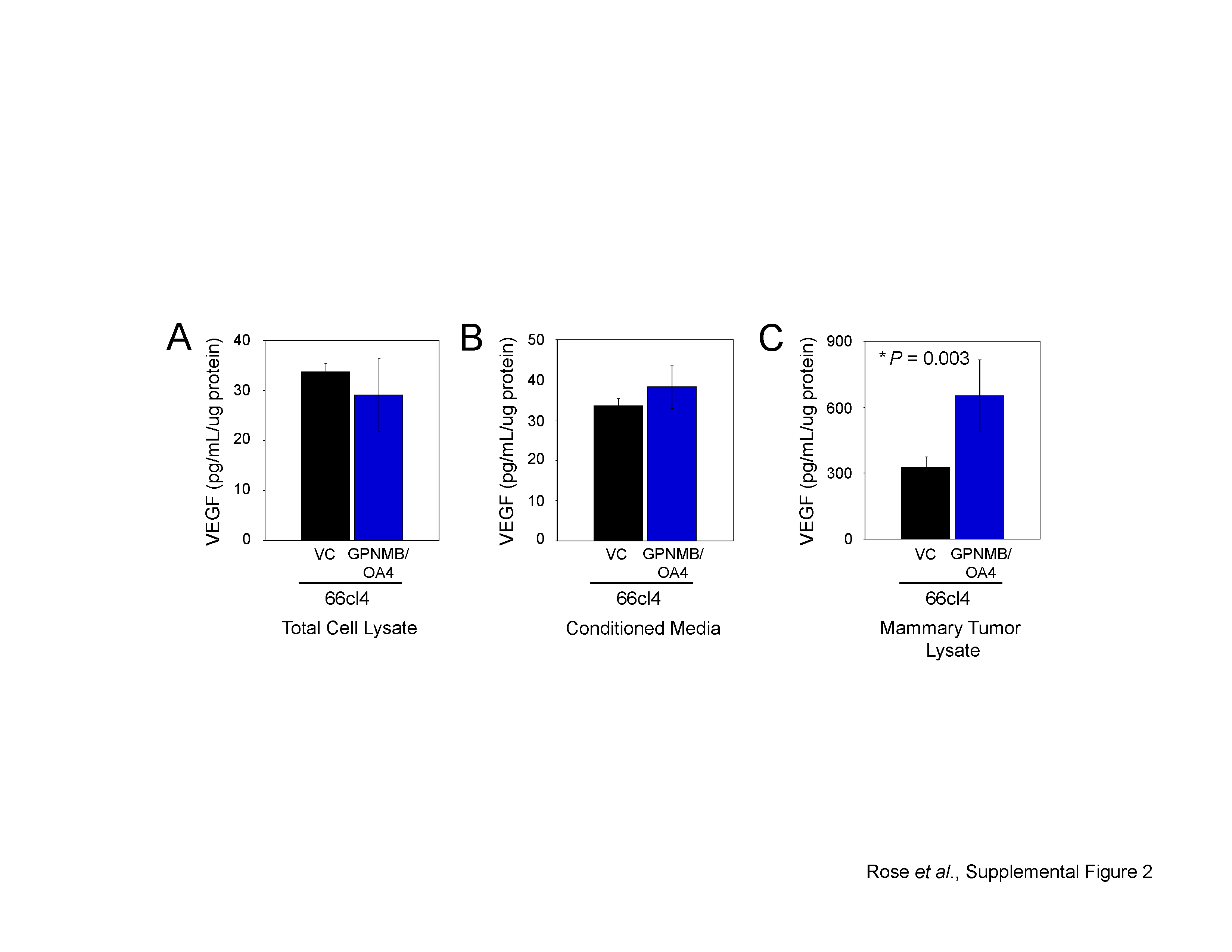

Supplement: Figure S2 — Analysis of VEGF expression and endothelial recruitment in breast cancer cells expressing GPNMB/OA. (A) Total cell lysates and (B) cell supernatants were extracted from vector control (VC, black bars) and GPNMB/OA-expressing (GPNMB/OA4, blue bars) 66cl4 cells grown in vitro and from (C) tumors grown in vivo. Tumors were excised at a volume of 200–300mm3 and flash frozen in liquid nitrogen. VEGF protein was quantified using ELISA and normalized to the total amount of protein in the corresponding cell lysate (A, B) or tumor lysates (C). *, P = 0.003, Students t-test. (0.52 MB TIF) [file pone.0012093.s002.tif]

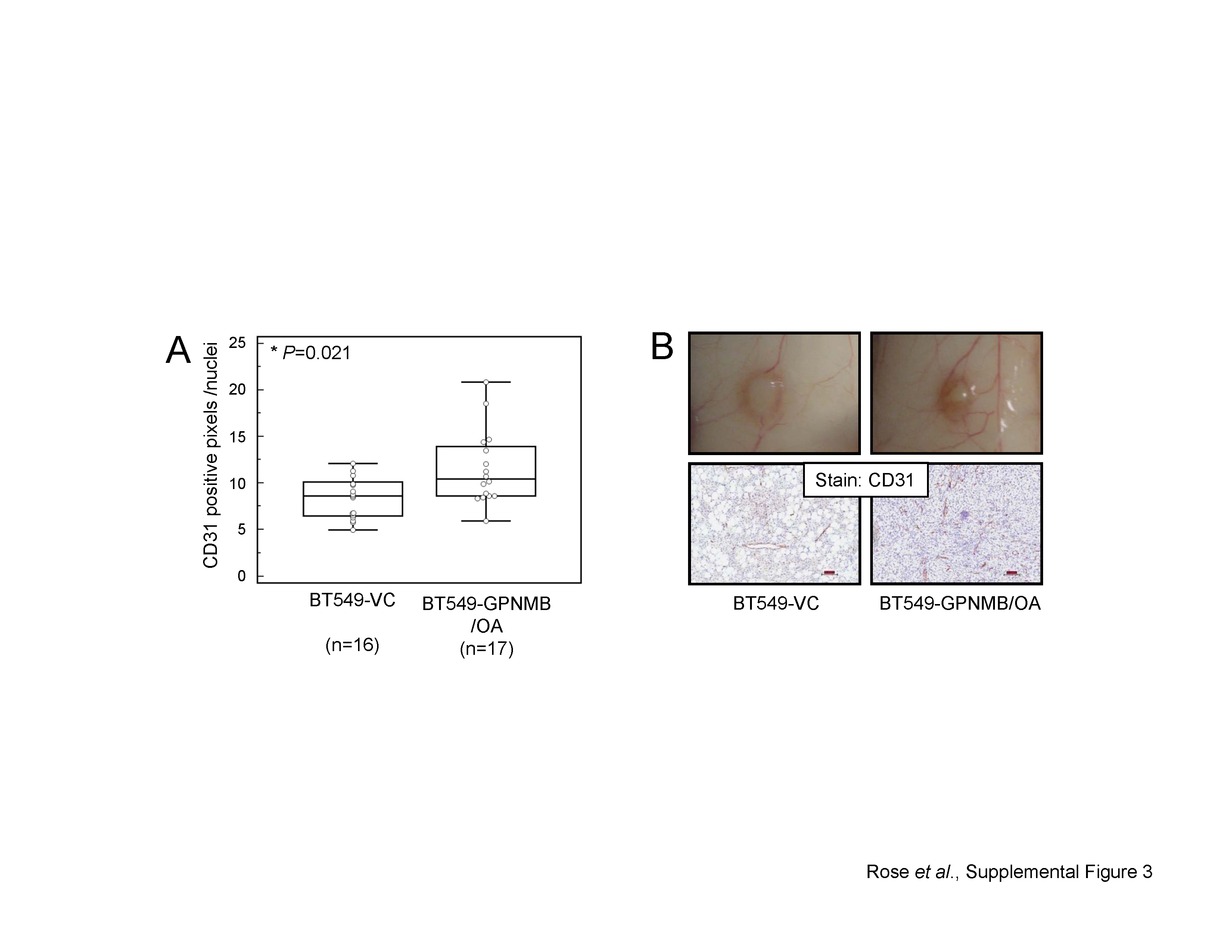

Supplement: Figure S3 — GPNMB/OA promotes angiogenesis in an in vivo human breast cancer model. VC or GPNMB/OA-expressing BT549 cells (1×10^6^) were suspended in a 50∶50 solution of PBS∶matrigel and injected subcutaneously into athymic mice and the animals sacrificed 10 days later. (A) CD31 (endothelial marker)-stained pixels were quantified for each matrigel plug and normalized to the number of total nuclei in the section. *, P = 0.021, Students t-test. (B) Vasculature recruited into the matrigel plugs was visualized on the inner surface of the skin (upper panels). Representative images of CD31 stains are shown (lower panels). Scale bars represent 100 µm. (1.96 MB TIF) [file pone.0012093.s003.tif]
